# The Study on Biological Function of *Keratin 26*, a Novel Member of Liaoning Cashmere Goat Keratin Gene Family

**DOI:** 10.1371/journal.pone.0168015

**Published:** 2016-12-20

**Authors:** Mei Jin, Jing Wang, Ming-Xing Chu, Jun Piao, Jing-Ai Piao, Feng-Qin Zhao

**Affiliations:** 1 Department of Life Sciences, Liaoning Normal University, Dalian, Liaoning, China; 2 Institute of Animal Sciences, Chinese Academy of Agricultural Sciences, Beijing, China; China Agricultural University, CHINA

## Abstract

In our research, we explored the relationship between *Keratin 26* and the regulation of fine hair, BMP signaling pathway, MT, FGF5, and IGF-I. The result of hybridization in situ revealed that *Keratin 26* was specially expressed in cortex of skin hair follicles; the result of immunohistochemistry indicated that Keratin 26 was expressed in internal root sheath, external root sheath. Then, Real-time quantitative PCR results showed that relative expressive quantity of *Keratin 26* was 1.08 or 3.3 × greater in secondary follicle than primary follicle during anagen or catagen; the difference during anagen was not remarkable (p>0.05), however, that of catagen was extremely significant (p<0.01). Relative expressive quantity of *Keratin 26* increased during telogen; the difference was extremely significant (p<0.01). Moreover, after *Noggin* expression interference using RNAi technology, we found that relative expressive quantity of *Keratin 26* extremely remarkably declined (p<0.01); after *K26* overexpression, we found that relative expressive quantity of *Noggin* extremely remarkably increased (p<0.01). We detected expressive quantity change of *Keratin 26* and Keratin 26 using Real-time quantitative PCR and immunofluorescence technologies after fibroblasts were treated with MT, FGF5 or IGF-I; the results indicated that MT and FGF5 played a positive role in *Keratin 26* and Keratin 26 expression, IGF-I played a negative role in *Keratin 26* expression, positive role in Keratin 26 expression. The results above showed that *Keratin 26* could inhibit cashmere growth, and was related to entering to catagen and telogen of hair follicles; *Keratin 26* and BMP signaling pathway were two antagonistic pathways each other which could inhibit growth and development of cashmere; MT, FGF5 and IGF-I could affect expression of *Keratin 26* and Keratin 26, and Keratin 26 was one of the important pathways that MT induced cashmere production in advance, FGF5 regulated cashmere growth and IGF-I promoted cashmere growth and development.

## Introduction

Goat cashmere grows in outer surface cortex of goat skin. Goat cashmer is very percious, its yield is rare and quality is excellent. Seventy percent of cashmere all over the world comes from China, and the quality of cashmere from China is superior to that from other countries. Liaoning cashmere goat that comes from Gaizhou located in Liaotung Peninsula is an excellent breed whose cashmere yield is the highest in China.

Many studies indicated that the keratin composition of cashmere was closely related to the cashmere quality, and the relationship could change when it was affected by heredity, nutrition, physiology, environment and other factors [1]. The expression location of keratin genes was detected before, *keratin 25-keratin 28*, *keratin 6irs1-keratin 6irs4* of human were specifically expressed in the internal root sheath of skin hair follicles [2]; *keratin 27*, *keratin 31*, *keratin 35*, *keratin 38 and keratin 85* of sheep were expressed in cortex and cuticle of skin hair follicles [3]. Then, many researches indicated that keratins played a very important role in growth process of hair, for example, *keratin 17 and tumor necrosis factor-α* could regulate growth cycle of hair [4]; keratin 2-keratin 5 were involved in growth process of hair [5]. In addition, some keratins were also related to the function of epithelial cells, for example, keratin 8 and keratin 18 could guarantee rigidity and structure integrity of extracellular matrix of epithelial cells, affect intracellular transportation mechanism and signaling pathway, and might inhibit the apoptosis of epithelial cells [6–8]; keratin 8 could play a role in infection activity of transferable cells of epithelium [9]. In addition, keratins also had other important function on cells, for example, keratin 18 played a significant role in estrogen receptor α signaling pathway regulation [10]; keratin 23 could regulate functional status of 14-3-3ε scaffolding protein in cytoplasm, and then regulate signal transduction, cell cycle, cell apoptosis and other processes [11]. In the early time, bioinformatics analysis and gene localization analysis of *keratin 26* in Liaoning cashmere goat were conducted by Jin Mei, Xing Mengxin and other people, and they found that the relationship between *keratin 26* expression and skin [12].

Hair follicle is an important structure that regulates cashmere growth, it is divided into primary and secondary follicles, primary follicles produce hair, secondary follicles produce cashmere. Many hormones and cytokines can regulate hair follicle growth and development, for example, melatonine (MT), fibroblast growth factor 5 (FGF5) and insulin-like growth factor-1 (IGF-I). MT is one kind of highly conserved indole hormone, it plays a very important role in many cells, tissues and organs [13], it is mainly secreted by pineal body, and skin is another important place of MT compound and metabolism besides pineal body [14–15]; some scholars found that MT might affect hair growth of human in the body through injecting MT locally [16]; other scholars made hair follicles enter into anagen from telogen earlier in contrast with control group through injecting MT into New Zealand goat everyday [17]; researchers found that exogenous MT could increase initial activity of secondary follicle growth and DNA synthesis, and then promote the hair shaft extension of secondary follicles through culture experiments in vitro [18]; in addition, exogenous MT could also promote mature cashmere loss quickly and new cashmere growth [19]. FGF5 is one kind of important regulatory factor of hair growth, the member of FGF gene family; researchers found that growth VI period of hair growth cycle extended after knockout of *FGF5* of rat, and then it led to elongating of clothing hair [20]; some scholars speculated that the missense mutation of *FGF5* resulted in long hair character of dog through the segregation experiment of autosome recessive inheritance of dog [21]; other scholars found that *FGF5* mutation was relate to the hair length [22]; in addition, researchers also found that *FGF5* product could regulate growth cycle of rat hair through synergy [23]. IGF-I is the important member of IGF family, it has extremely important and rich biological function as one kind of bioactive polypeptides in the body; some researches indicated that IGF-I played an important role in transition and activation of hair follicle cycle [24], and could regulate growth development and cycle of hair follicle [25–26]; other scholars found that IGF-I could stimulate hair follicle growth dramatically [26], and the combination of IGF-I and the corresponding receptors could regulate growth and development of skin hair follicle, and then affect the fineness and length of wool [27–28].

In 1965, Urist and other people found bone morphogenetic proteins (BMPs) [29], more than 20 members of BMP family were found until now [30]; BMP ligand need to specifically combine specific receptor located in the target cell membrances, the corresponding receptor of BMP ligand is divided into 3 subtypes—bone morphogenetic protein receptor IA (BMPRIA), BMPRIB, and BMPRII [31], BMP signaling pathway is mediated by BMPRI and BMPRII together; BMP combines BMPRII first, then the complex combines BMPRI, BMPRI is activated, and then the downstream effector molecules are recruited, effector molecules activated transport into cell nucleus, transcriptional complex will form under the the synergistic action of other transcription factors and combine regulatory region of target gene, and then target gene expression and development of biological effect will be regulated [32]; there are 4 pathways that BMP regulates steady state and development of cuticle and hair follicle—BMP-Smad pathway, BMP-mitogen-activated protein kinase (BMP-MAPK) pathway, BMP-Wnt pathway, Edar-BMP pathway, Edar-BMP signaling pathway is also related to the production of primary follicles [33–35]. The research indicated that types, quantity, locations of BMP and BMPR expression were different in the hair follicle cycle [36]; BMP signaling pathway could affect dermal papilla growth, hair types and fiber fineness during anagen through regulating the reproduction of keratinocyte to some extent [33, 37]; other research showed that BMP, Wnt signaling pathway and GATA-3 could affect the bulb cell formation of inner root sheath, and then increase cashmere yield [38]; BMP signaling pathway could also regulate development of ectodermal organs to some extent [39], in addition, BMP signaling pathway could regulate the beginning of anagen, be involved in hair follicle cell apoptosis and inhibit hair follicle formation [40–41]. There were many antagonists of BMP signaling pathway outside the cell, among all antagonists, Noggin was the most typical in regulatory effect respect, it could competitively inhibit the combination between BMP and BMPR, and then inhibit BMP signaling pathway [42]; the experiment indicated that hair follicle formation delayed after *Noggin* of rat skin knockout, on the contrary, Noggin could stimulate hair follicle formation after adding exogenous Noggin, therefore the researchers considered that hair follicle formation of skin during embryonic period was associated with the antagonism of Noggin to BMP4 [43]; in addition, some researchers found that, if *BMP2* expression in chicken embryo was abnormal, hair follicle germ formation would be inhibited, on the contrary, if *Noggin* was deleted, the induction of secondary follicle formation would stop completely and primary follicle morphogenesis was normal, according to the above, Noggin was an important factor during formation process of secondary follicles, and the effect of Noggin might be realized through affecting BMP2 [44]; Noggin could induce and activate the hair follicles during telogen in vitro, but BMP4 could make the anagen of secondary follicle stop, it illustrated that Noggin could regulate the anagen induction of postnatal skin hair follicle through regulating BMP4 [45].

According to previous researches, we found that many keratins in human and sheep all were expressed in skin hair follicles and closely related to hair growth, BMP signaling pathway also was closely associated with hair follicle development and cashmere growth, those indicated there was some relationship between keratins and BMP signaling pathway, we need to further study and confirm it.

Now, there are more and more researches about keratins, but there is few research about the keratins of cashmere goat. In our study, we analyzed *keratin 26* and keratin26 expression qualitatively and quantitatively first, then we explored the relationship between keratin 26 and BMP signaling pathway through gene interference and overexpression technologies, finally, we analyzed the effect of MT, FGF5 and IGF-I on *keratin 26* and keratin 26 by quantitative real-time polymerase chain reaction (PCR) and immunofluorescence technologies quantitatively. These results will establish the theoretical basis of exploring the regulatory mechanism of cashmere quality and yield, and provide the theoretical basis for breeding excellent cashmere goat breed.

## Materials and Methods

The study protocol was approved by the ethics committee of Liaoning Normal University.

### In situ hybridization

First, Liaoning cashmere goat (It came from Liaoning cashmere goat breeding station and was a ram. It was one year old and healthy. It was raised in a 3 × 3 square meters indoor feeding room at 25°C. We cleaned up the room, brought the goat out for 30 min once a day and bathed it once every two weeks. In addition, we provided a basin of fresh running water and enough fresh grass every day.) skin samples in anagen (in October) were collected (We collected the sample of 1 × 1 square centimeters from the trunt of the goat, and we usually transferred its attention, helped it relax and eased its pain through feeding grass and combing its cashmere). Next, 104.5 ng/μL keratin 26 cDNA was used as digoxin-labeled template, and the probe was prepared based on instructions provided by the Digoxin Labeling Kit (Roche Company). After the skin slices were dewaxed, rehydrated, and digested by protease K, desiccated and so on, prehybridization of the slice in 50% deionized formamide was carried out for 1 h in the 20 × SSC (Sodium citrate buffer solution) wet box, and prehybridization was carried out overnight at 42°C, and then the slice was washed off in 2 × SSC, 1 × SSC, and 0.25 × SSC wet box and diluted with antibody (1:5000). After binding to antibody, the slice was dyed using NBT/BCIP (Nitroblue tetrazolium/5-Bromo-4-chloro-3-indolyl phosphate) and colored using eosin. Finally, the slice was sealed using resinene, baked, and pictures were taken.

### Immunohistochemistry

First, Liaoning cashmere goat (from Liaoning cashmere goat breeding station) skin samples in anagen (in October) were collected. Next, skin samples were paraffinized, and 4 μm paraffin sections were baked in a 50–60°C incubator for 30–60 min, dewaxed to water, incubated for 5–15 min using 3% H_2_O_2_ deionized water, washed using distilled water, soaked for 5 min (three times) using PBS (Phosphate buffered saline), wiped dry, soaked in sodium citrate buffer solution (pH = 6), baked on medium-high and medium-low in a microwave oven for 6 min respectively, then cooled naturally to room temperature. After cooling, the sections were soaked for 3 min (three times) using PBS, sealed using normal goat serum for blocking for 10–30 min at room temperature, incubated for 24 h at 4°C using primary antibody (PBS was used instead of primary antibody in the control group), rewarmed for 20–30 min, washed for 5 min (five times) using PBS, incubated for 30–60 min at 37°C using biotin-labeled second antibody, washed for 5 min (five times) using PBS, incubated for 30–60 min at 37°C using SP (Spectroscopic pure), washed for 5 min (five times) using PBS, colored for a few seconds to 1 min using diaminobenzidine, adequately washed for 10 min using running water, overdyed for a few seconds to 1 min with hematoxylin, washed adequately for 10 min using running water, and dehydrated until the sections were lucency. Finally, the paraffin section was sealed using resinene, baked, and pictures were taken.

### Quantitative real-time PCR

First, Liaoning cashmere goat (from Liaoning cashmere goat breeding station) skin samples in anagen (in October), catagen (in February), and telogen (in June) were collected and separated into primary and secondary follicles. Next, total RNA of primary and secondary follicles during anagen and catagen, and skin during telogen were extracted using the Trizol Kit (TaKaRa Company). The total RNA extracted was treated with DNAse I, RNA was reverse transcribed into cDNA according to the instructions in the RT-PCR Kit (TaKaRa Company). Finally, quantitative real-time PCR was carried out, and experiments were repeated three times for each sample, with *β-Actin* as the reference gene, primer sequences was shown in Table 1, reaction system was shown in Table 2, and reaction condition was detailed in Table 3. Experiment conditions, such as annealing temperature and primer concentrations, were optimized by standard PCR prior to conducting the experiments.

**Table 1:** Gene names and primer sequence.

Gene name	Primer sequence (5’---3’)	Length	Tm	GC %	Product Length
***β-Actin***	(F) CCAAAGCCAACCGTGAGAA	19	58.29	52.63	101 bp
***β-Actin***	(R) AGAGGCGTACAGGGACAGCA	20	63.07	60.00	
***keratin 26***	(F) CACTGGTCGGCTAACTGG	18	57.40	61.11	1822 bp
***keratin 26***	(R) CACGCCCTTCTGATTTGT	18	55.25	50.00	
***Noggin***	(F) GTATGCGTGGAACGACCTGG	20	61.08	60.00	205 bp
***Noggin***	(R) GGAAATGATGGGGTACTGGATG	22	58.51	50.00	

**Table 2:** Reaction systems of quantitative real-time PCR.

Component	Volume (*keratin 26*)	Volume (*Noggin*)
**SYBR Premix Ex Taq II (2×)**	10 μL	10 μL
**Primer F**	0.4 μL	0.5 μL
**Primer R**	0.4 μL	0.5 μL
**RT-PCR product**	1 μL	1 μL
**dH**_**2**_**O**	8.2 μL	8 μL
**Total**	20 μL	20 μL

**Table 3:** Reaction conditions of quantitative real-time PCR.

Cycle number	Temperature (*keratin 26*)	Time (*keratin 26*)	Temperature (*Noggin*)	Time (*Noggin*)
**1**	95	10 sec	95	2 min
**40**	95	5 sec	95	15 sec
	60	30 sec	59	20 sec
	72	—	72	—

### *Noggin* expression interference effect on *keratin 26* expression

First, Liaoning cashmere goat (from Liaoning cashmere goat breeding station) skin samples in anagen (in October) were collected. Next, the samples were cultivated using DMEM (Dulbecco ‘s modified eagle medium) + 20% FBS (Fetal bovine serum) + 1% P/S (Penicillin/Streptomycin) + 1% glutamax in a CO_2_ incubator at 37°C with 5% CO_2_/95% air.

In the preliminary experiment, the best MOI (Multiplicity of infection) value for lentivirus-infected Liaoning cashmere goat fibroblasts was detected using negative virus liquid. The fibroblasts in the logarithmic phase were digested using pancreatin and prepared into cell suspension, the cell suspension was inoculated into 12-well plates and cultivated in a CO_2_ incubator at 37°C with 5% CO_2_/95% air until cell confluence reached approximately 80%; the MOI gradient was set to 0, 1, 10, 30, 50, and 100, and corresponding doses of negative virus liquid were added into the cell suspension according to the virus titer, meanwhile 5 μg/mL polybrene, which is a kind of dyeing assistant, was added into the cell suspension to enhance infection effect, the cells were cultivated in complete medium, which had no virus and polybrene, replaced the previous medium after viral infection for 12 h, GFP (Green fluorescent protein) expression in each well was observed using a fluorescence microscope after viral infection for 72 h, the infection efficiency of each group was pictured and recorded to ensure the best MOI value that infection efficiency reached above 80%.

Then, the *Noggin*-interfering lentivirus and lentivirus to be used as negative controls infected Liaoning cashmere goat fibroblasts. The best MOI value that lentivirus infected Liaoning cashmere goat fibroblasts was 30 according to the results in the preliminary experiment, *Noggin*-interfering lentivirus and lentivirus to be used as negative controls were added into the cell suspension respectively according to virus titer, meanwhile 5 μg/mL polybrene was added into the cell suspension to enhance the infection effect; the cells were cultivated in complete medium, which had no virus and polybrene, replaced the previous medium after viral infection for 12 h; GFP expression in each well was observed using a fluorescence microscope after viral infection for 7 d, the associated results were pictured and recorded, if the fluorescent rate was not greater than 80%, the experiment was re-run.

Total RNA of fibroblasts that was virally infected were extracted using the Trizol Kit (TaKaRa Company), RNA was reverse transcribed into cDNA according to the instructions in the M-MLV Kit (Fermentas Company), and *Noggin* expression after *Noggin*-interfering lentivirus infected Liaoning cashmere goat fibroblasts was detected by quantitative real-time PCR, primer sequences was shown in Table 1, reaction system was shown in Table 2, and reaction condition were described in Table 3, *keratin 26* expression after the *Noggin*-interfering lentivirus infected primary skin cells was detected by quantitative real-time PCR.

### *keratin 26* overexpression effect on *Noggin* expression

First, Liaoning cashmere goat (from Liaoning cashmere goat breeding station) skin samples in anagen (in October) were collected. Next, the samples were cultivated with DMEM + 20% FBS + 1% P/S + 1% glutamax in a CO_2_ incubator at 37°C with 5% CO_2_/95% air. Then, *keratin 26*-overexpressing lentivirus and lentivirus to be used as negative controls infected Liaoning cashmere goat fibroblasts. The skin cells that were in the logarithmic phase were digested using pancreatin and prepared into cell suspension, the cell suspension was inoculated into 6-well plates and cultivated in a CO_2_ incubator at 37°C with 5% CO_2_/95% air until cell confluence reached approximately 80%; the best MOI value that lentivirus infected Liaoning cashmere goat fibroblasts was 30, *keratin 26*-overexpressive lentivirus and lentivirus to be used as negative controls were added into the cell suspension respectively according to virus titer, meanwhile 8 μg/mL polybrene was added into the cell suspension to enhance the infection effect of infection; the cells were cultivated in complete medium, which had no virus and polybrene, replaced the previous medium after viral infection for 8 h. GFP expression in each well was observed using a fluorescence microscope after viral infection for 72 h, the associated results were pictured and recorded, if the fluorescent rate was not greater than 80%, the experiment was re-run.

Total RNA of virus-infected fibroblasts was extracted using the Trizol Kit (TaKaRa Company), RNA was reverse transcribed into cDNA according to the instructions in the M-MLV Kit (Fermentas Company), and *keratin 26* expression after *keratin 26*-overexpression lentivirus infected Liaoning cashmere goat fibroblasts was detected by quantitative real-time PCR, finally, *Noggin* expression after *keratin 26*-overexpression lentivirus infected fibroblasts was detected by quantitative real-time PCR.

### Quantitative real-time PCR detection after MT, FGF5, IGF-I, MT + FGF5, and MT + IGF-I treatment of Liaoning cashmere goat fibroblasts

First, Liaoning cashmere goat (from Liaoning cashmere goat breeding station) skin samples in anagen (in October) were collected. Next, Liaoning cashmere goat fibroblasts were primary cultured. The tissue blocks were washed two to three times using D-Hanks solution and cut into 1 mm^3^ patches; the patches were placed 0.5 cm apart at the bottom of the culture bottles and cultured in a CO_2_ incubator at 38.5°C with 5% CO_2_/95% air for 2 h to slightly dry the patches; then, 10 mL DMEM was injected into the bottom of the bottles after adherence of the patches, and the patches were cultured in an incubator for 2–3 d, the culture solution was added into the bottles when the cell quantity that were swum out from the tissue blocks increased.

Then, the Liaoning cashmere goat fibroblasts were subcultured. When cell confluence reached 70%-80%, the culture solution was removed; the cells were washed twice with DBPS (Dulbecco phosphate-buffered saline), cultured for 3–5 min in an incubator at 38.5°C after adding 3 mL of preheated 0.25% trypsin, and observed with a microscope; when 80% of the cells fell from the bottle wall, became round, and separated into individual cells, the bottles were patted to make all cells fall from the bottle wall; 6 mL of DMEM culture solution that contained 10% FBS was injected into the bottles, the mixture was centrifuged for 5 min at 1000 r/min, supernatant was discarded, culture solution was added into the bottles, cells were resuspended and inoculated into new culture bottles and cultured, culture solution was changed after 2 d; the cells were subcultured after the cells adhered to the wall for 24 h and cell confluence reached 70%-80%.

Liaoning cashmere goat fibroblasts were first treated with MT, FGF5, or IGF-I respectively, the fibroblasts were treated with MT, FGF5, or IGF-I in concentrations of 0 g/L, 0.02 g/L, 0.2 g/L, 1 g/L MT or 0 g/L, 10^−6^ g/L, 10^−5^ g/L, 10^−4^ g/L FGF5 or 0 g/L, 10^−6^ g/L, 10^−5^ g/L, 10^−4^ g/L IGF-I, the concentrations of cells in each group were all 10^5^ /mL, and cells were cultured for 24 h, 48 h, or 72 h respectively. Next, fibroblasts were treated with combinations of MT (1 g/L) and FGF5 (10^−5^ g/L) or MT (1 g/L) and IGF-I (10^−5^ g/L) for 72 h.

Total RNA of the fibroblasts in each group was extracted using the Trizol Kit (TaKaRa Company), the total RNA extracted was treated with DNAase I, RNA was reverse transcribed into cDNA according to the instructions provided in the RT-PCR Kit (TaKaRa Company), and quantitative real-time PCR was carried out.

### Immunofluorescence detection after MT, FGF5, and IGF-I treatment of Liaoning cashmere goat fibroblasts

First, Liaoning cashmere goat (from Liaoning cashmere goat breeding station) skin samples in anagen (in October) were collected. Next, the fibroblasts were primary cultured and subcultured.

The optimal conditions under which MT, FGF5, and IGF-I affected keratin 26 expression were detected using western blot in the preliminary experiment. The cell suspension was inoculated into 6-well plates and cultivated in a CO_2_ incubator at 37°C with 5% CO_2_/95% air, the cells were then pictured and recorded; the fibroblasts were treated with 0.02 g/L, 0.2 g/L, 1 g/L MT or 10^−6^ g/L, 10^−5^ g/L, 10^−4^ g/L FGF5 or 10^−6^ g/L, 10^−5^ g/L, 10^−4^ g/L IGF-I at cell concentrations of 10^5^ /mL, and cells were cultured for 24 h, 48 h, or 72 h, the cultured cells were pictured and recorded; total proteins were extracted from the treated cells, and SDS-PAGE (Sodium dodecylsulfate-polyacrylamide gel electrophoresis) was carried out; after SDS-PAGE, western blot was carried out; finally, immunofluorescence was carried out, the results were pictured and recorded, and gray analysis of the result maps was carried out to determine optimal concentration and culture time for MT, FGF5, and IGF-I to affect keratin 26 expression.

Liaoning cashmere goat fibroblasts were first treated with MT, FGF5, or IGF-I respectively. The cell suspension was inoculated into 6-well plates and cultivated overnight in a CO_2_ incubator at 37°C with 5% CO_2_/95% air; the culture solution was changed the next day, the cells were treated with 0.2 g/L MT, 10^−5^ g/L FGF5, or 10^−6^ g/L IGF-I, the treated concentrations were selected according to the results of the preliminary experiment; the cells were pictured and recorded after treatment for 72 h and collected to prepare cell suspension, the cell suspension was inoculated into 24-well plates and cultivated overnight in a CO_2_ incubator at 37°C with 5% CO_2_/95% air.

Immunofluorescence analysis was carried out. The creep plates inoculated cells were carefully removed from 24-well plates using a pincette after overnight, placed on glass slides, then marked; the cells were fixed for 15 min using 4% paraformaldehyde, washed two times using PBS, treated for 10 min using 0.1% Triton-100 prepared with PBS to increase cell membrane permeability, sealed using confining liquid prepared with 4% BSA (Bovine serum albumin) and PBST (Phosphate buffer solution twain) for 1 h at room temperature; primary antibody was diluted using fresh confining liquid, the cells were incubated using primary antibody overnight at 4°C, and washed five times with PBS; the second antibody that corresponded to the primary antibody with FITC (Fluorescein isothiocyanate) labeling was diluted using fresh confining liquid, the cells were incubated for 1 h at room temperature with the second antibody, and washed five times with PBS; the cell nuclei were stained for 10 min using DAPI (4´,6-diamidino-2-phenylindole), and the cells were washed five times with PBS; excess moisture in the creep plates was absorbed, and the creep plates were sealed using sealing liquid resisting to cancellation, then, the cells were observed by confocal microscopy, pictured and recorded, and gray analysis of the result maps was carried out.

## Results

### Location and expression of *keratin 26* and keratin 26

**Fig 1:**
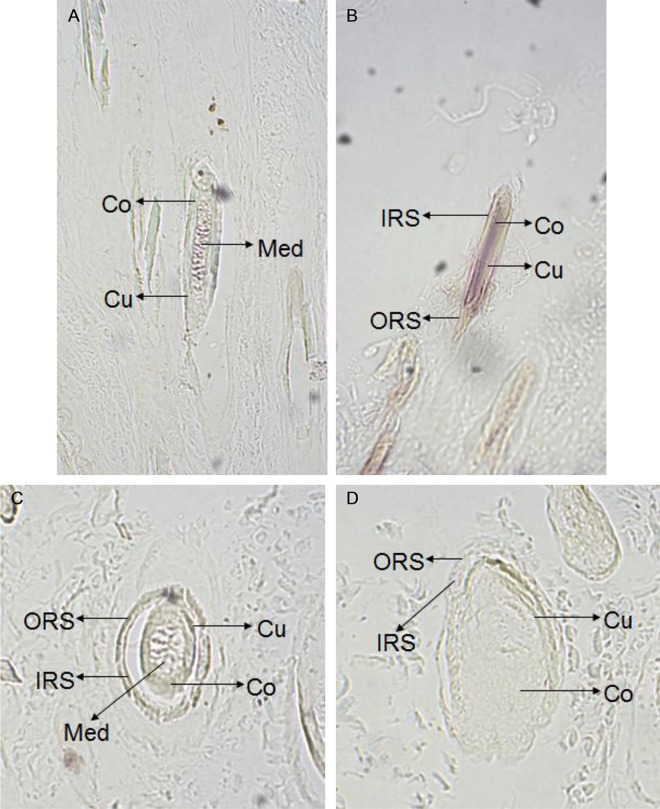
Expression of *keratin 26* in skin was detected by in situ hybridization in experimental group (100×). Fig 1A. Longisection of primary follicle in experimental group. Fig 1B. Longisection of secondary follicle in experimental group. Fig 1C. Transection of primary follicle in experimental group. Fig 1D. Transection of secondary follicle in experimental group.

**Fig 2:**
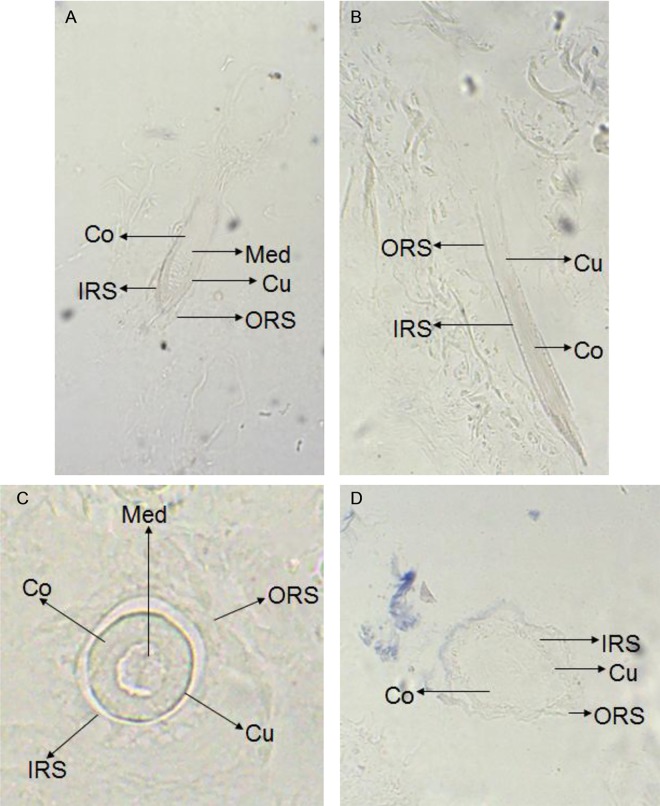
Expression of *keratin 26* in skin was detected by in situ hybridization in control group (100×). Fig 2A. Longisection of primary follicle in control group. Fig 2B. Longisection of secondary follicle in control group. Fig 2C. Transection of primary follicle in control group. Fig 2D. Transection of secondary follicle in control group.

**Fig 3:**
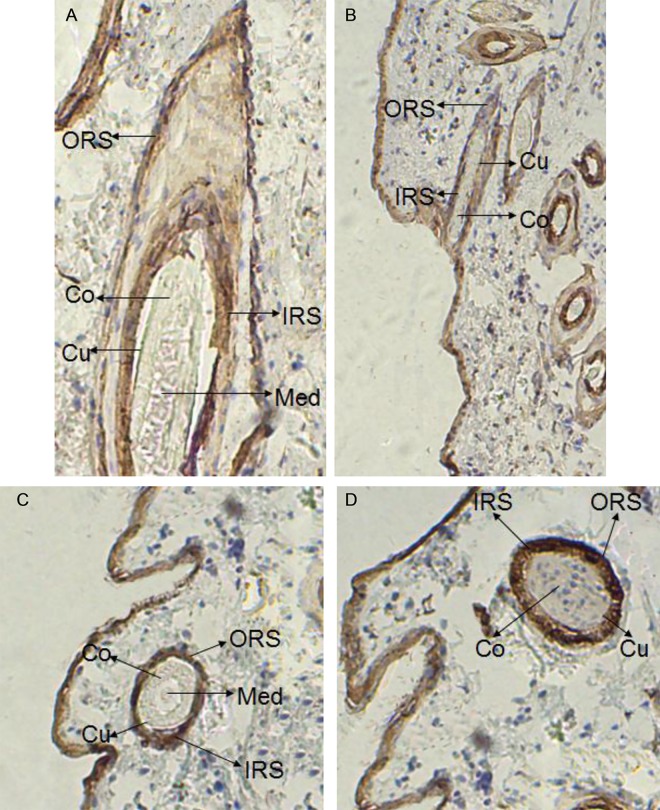
Expression of keratin 26 in skin was detected by immunohistochemistry in experimental group (100×). Fig 3A. Longisection of primary follicle in experimental group. Fig 3B. Longisection of secondary follicle in experimental group. Fig 3C. Transection of primary follicle in experimental group. Fig 3D. Transection of secondary follicle in experimental group.

**Fig 4:**
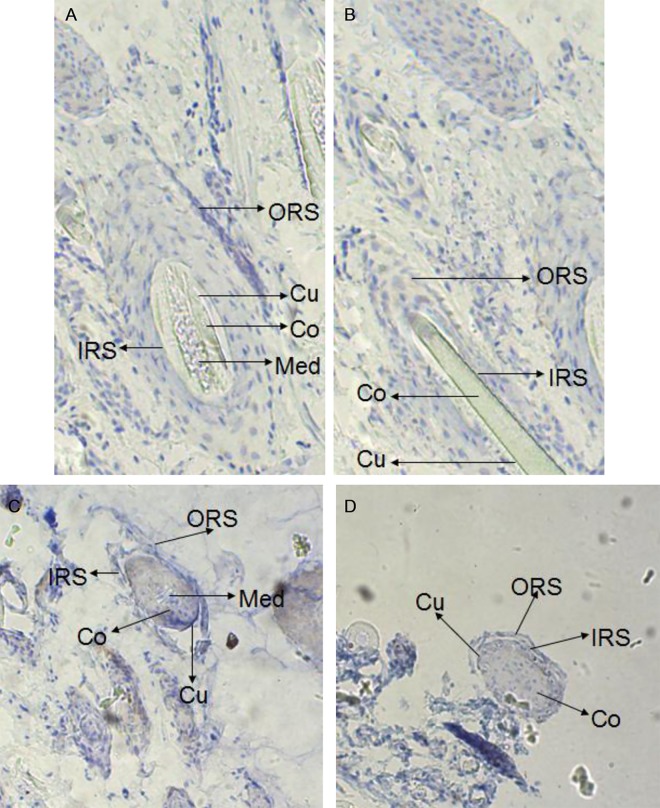
Expression of keratin 26 in skin was detected by immunohistochemistry in control group (100×). Fig 4A. Longisection of primary follicle in control group. Fig 4B. Longisection of secondary follicle in control group. Fig 4C. Transection of primary follicle in control group. Fig 4D. Transection of secondary follicle in control group.

In situ hybridization experiment results showed that there were weak expression signal in cortex layer of skin, no expression signal in medulla layer, outer root sheath and other structure of skin (Fig 1), and there was also no expression signal in blank control group (Fig 2), thus *keratin 26* is specifically expressed in cortex layer of skin. Immunohistochemistry experiment results indicated that there was expression signal in inner root sheath and outer root sheath (Fig 3), no expression signal in blank control group (Fig 4), therefore keratin 26 is specifically expressed in inner root sheath and outer root sheath.

**Fig 5:**
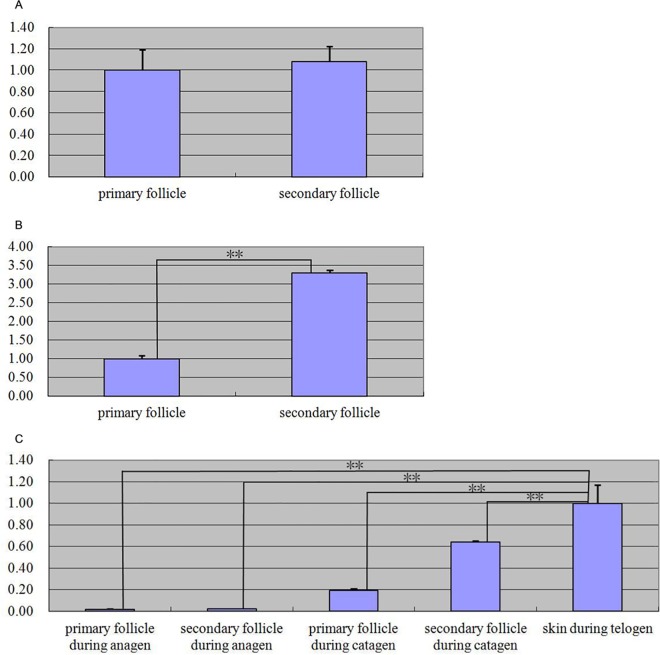
Expression quantity of *keratin 26* was detected during anagen, catagen and telogen by quantitative real-time PCR. Fig 5A. Relative *keratin 26* expression quantity was 1.08 × greater in secondary follicles than in primary follicles in anagen (p > 0.05). Fig 5B. Relative *keratin 26* expression quantity was 3.3 × greater in secondary follicles than in primary follicles in catagen (p < 0.01.). Note: * p < 0.05, ** p < 0.01. Fig 5C. Relative *keratin 26* expression quantity was 0.017884 ×, 0.019129 ×, 0.19 × and 0. 64 × less in primary and secondary follicles in anagen and catagen than in skin during telogen (p < 0.01).

Quantitative real-time PCR experiment results showed that *keratin 26* relative expression quantity was 1.08 × and 3.3 × greater in secondary follicles than primary follicles during anagen and catagen (Fig 5A and 5B), the difference of anagen was not significant (p > 0.05), but the difference of catagen was extremely significant (p < 0.01); because primary and secondary follicles couldn’t be distinguished clearly during telogen of hair follicles, we detected *keratin 26* expression of skin, the results indicated that *keratin 26* relative expression quantity during this period was higher than that of anagen and catagen periods (Fig 5C), the difference was all extremely significant (p < 0.01).

### *Noggin* expression interference and *keratin 26* overexpression

**Fig 6:**
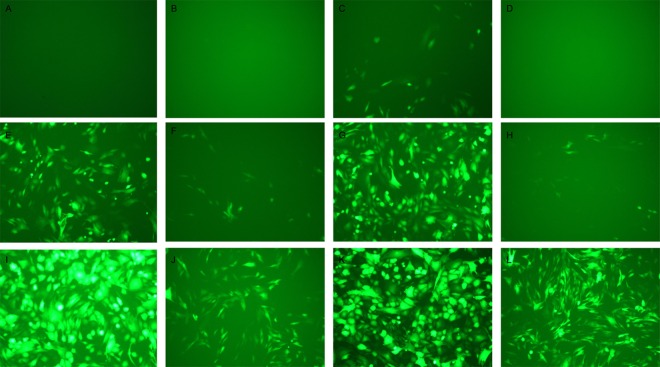
Skin cells which were infected with lentivirus for 72 h were observed under bright field of the fluorescence microscope in the preliminary experiment. Fig 6A. Skin cells which were added polybrene when MOI was 0. Fig 6B. Skin cells which were not added polybrene when MOI was 0. Fig 6C. Skin cells which were added polybrene when MOI was 1. Fig 6D. Skin cells which were not added polybrene when MOI was 1. Fig 6E. Skin cells which were added polybrene when MOI was 10. Fig 6F. Skin cells which were not added polybrene when MOI was 10. Fig 6G. Skin cells which were added polybrene when MOI was 30. Fig 6H. Skin cells which were not added polybrene when MOI was 30. Fig 6I. Skin cells which were added polybrene when MOI was 50. Fig 6J Skin cells which were not added polybrene when MOI was 50. Fig 6K. Skin cells which were added polybrene when MOI was 100. Fig 6L. Skin cells which were not added polybrene when MOI was 100.

**Fig 7:**
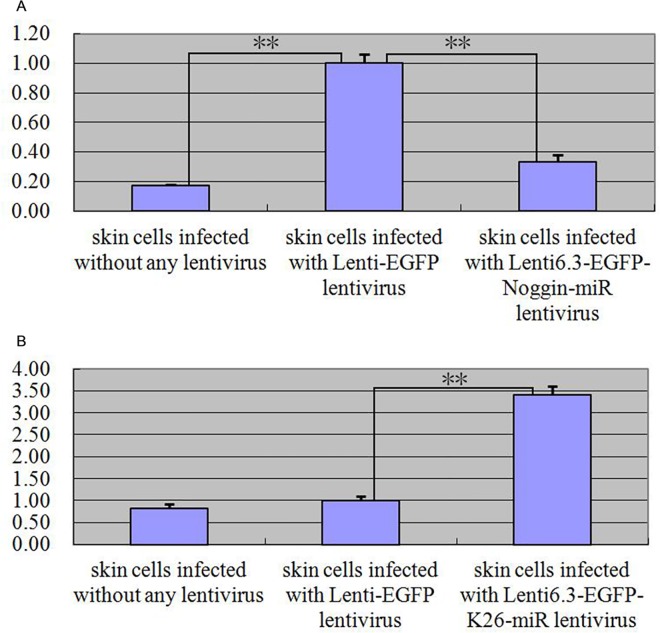
Expression quantity change of *keratin 26* after *Noggin* expression interference and *Noggin* after *keratin 26* overexpression was detected by quantitative real-time PCR. Fig 7A. Expression quantity of *keratin 26* was 0.17 × (p < 0.01), 0.33 × (p < 0.01) less in blank control group and experimental group than in negative control group. Fig 7B. Expression quantity of *Noggin* was 0.81 × (p > 0.05) less, 3.41 × (p < 0.01) greater in blank control group and experimental group than in negative control group.

In the preliminary experiment detecting the best multiplicity of infection (MOI) value that lentivirus affected primary skin cells of goat, we can found that the infection effect in the group adding polybrene was better than that of non-polybrene group, however, polybrene also could inhibit cell reproduction and make cell deform, thus the best MOI value that lentivirus affected primary skin cells of goat was 30, and adding polybrene would help heighten infection effect (Fig 6). After *Noggin* expression interference, quantitative real-time PCR detection result showed that *keratin 26* relative expression quantity declined (Fig 7A), and the expression quantity change was extremely significant (p < 0.01). After *keratin 26* overexpression, quantitative real-time PCR detection result indicated that *Noggin* relative expression quantity increased (Fig 7B), and the expression quantity change was also extremely significant (p < 0.01).

### MT, FGF5, and IGF-I effects on expression of *keratin 26* and keratin 26

**Fig 8:**
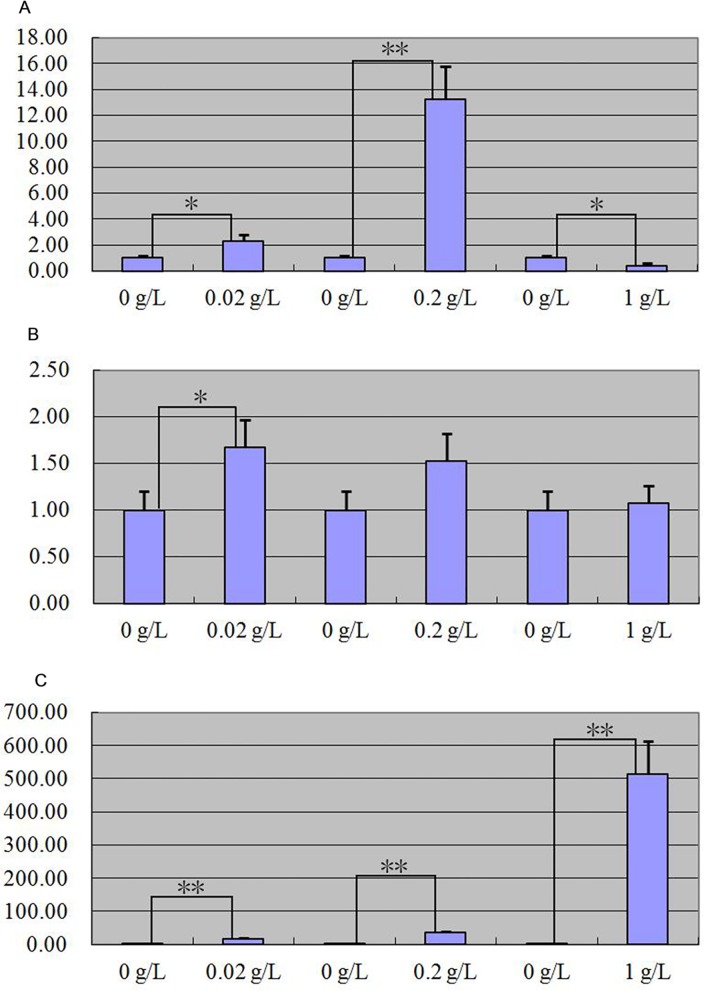
Expression quantity change of *keratin 26* after MT treatment respectively was detected by quantitative real-time PCR. Fig 8A. Expression quantity of *keratin 26* was 2.31 × (p < 0.05), 13.21 × (p < 0.01) greater, 0.41 × (p < 0.05) less in the groups which cells were treated with 0.02 g/L, 0.2 g/L, 1 g/L MT than the group which cells were treated with 0 g/L MT for 24 h. Fig 8B. Expression quantity of *keratin 26* was 1.67 × (p < 0.05), 1.52 × (p > 0.05) and 1.07 × (p> 0.05) greater in the groups which cells were treated with 0.02 g/L, 0.2 g/L, 1 g/L MT than the group which cells were treated with 0 g/L MT for 48 h. Fig 8C. Expression quantity of *keratin 26* was 15.99 × (p < 0.01), 35.15 × (p < 0.01), 513.36 × (p < 0.01) greater in the groups which cells were treated with 0.02 g/L, 0.2 g/L, 1 g/L MT than the group which cells were treated with 0 g/L MT for 72 h.

**Fig 9:**
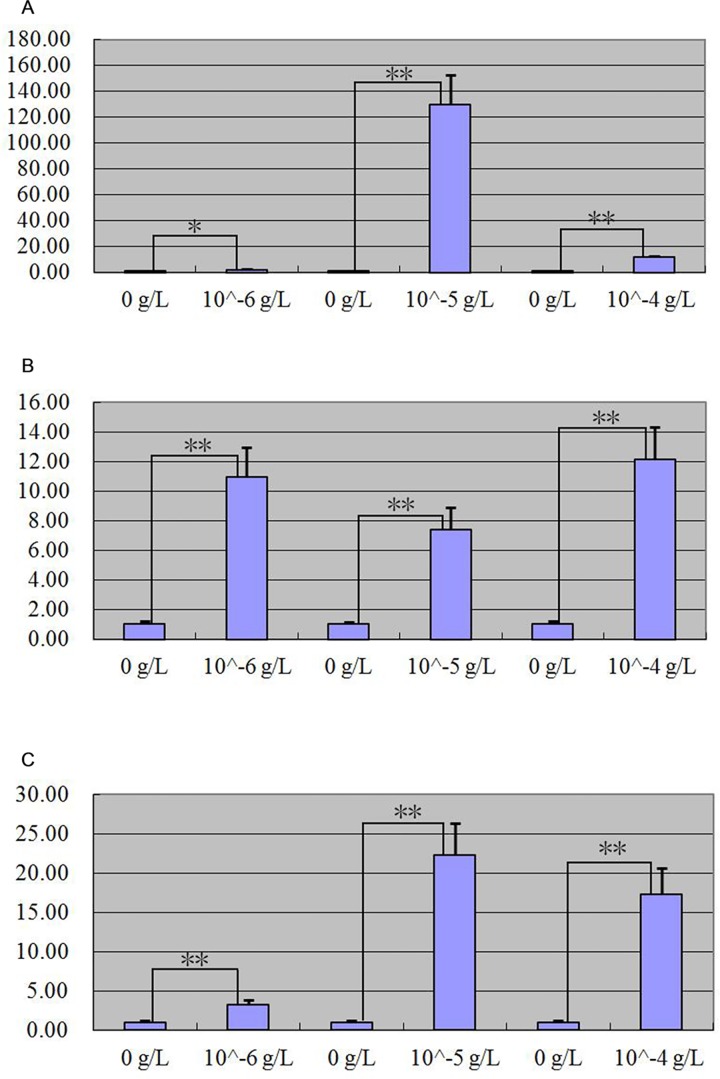
Expression quantity change of *keratin 26* after FGF5 treatment respectively was detected by quantitative real-time PCR. Fig 9A. Expression quantity of *keratin 26* was 1.88 × (p < 0.05), 129.91 × (p < 0.01), 11.95 × (p < 0.01) greater in the groups which cells were treated with 10^−6^ g/L, 10^−5^ g/L, 10^−4^ g/L FGF5 than the group which cells were treated for 0 g/L FGF5 for 24 h. Fig 9B. Expression quantity of *keratin 26* was 10.93 × (p < 0.01), 7.42 × (p < 0.01), 12.11 × (p < 0.01) greater in the groups which cells were treated with 10^−6^ g/L, 10^−5^ g/L, 10^−4^ g/L FGF5 than the group which cells were treated for 0 g/L FGF5 for 48 h. Fig 9C. Expression quantity of *keratin 26* was 3.21 × (p < 0.01), 22.3 × (p < 0.01), 17.31 × (p < 0.01) greater in the groups which cells were treated with 10^−6^ g/L, 10^−5^ g/L, 10^−4^ g/L FGF5 than the group which cells were treated for 0 g/L FGF5 for 72 h.

**Fig 10:**
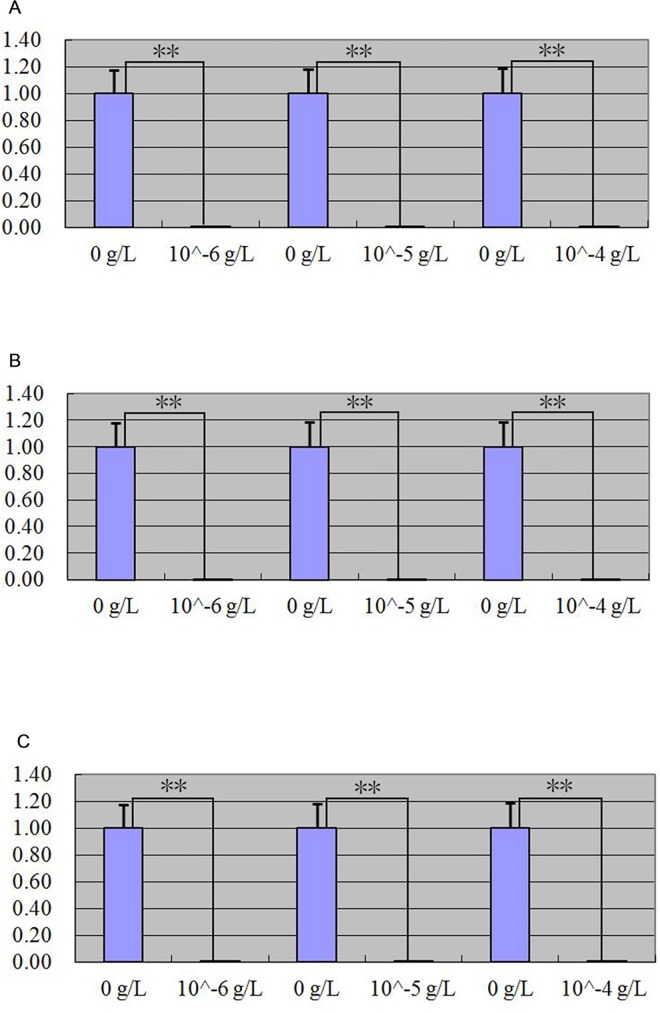
Expression quantity change of *keratin 26* after IGF-I treatment was detected by quantitative real-time PCR. Fig 10A. Expression quantity of *keratin 26* was 0.00011595 × (p < 0.01), 0.0000083 × (p < 0.01), 0.00004491 × (p < 0.01) less in the groups which cells were treated with 10^−6^ g/L, 10^−5^ g/L, 10^−4^ g/L IGF-I than the group which cells were treated for 0 g/L IGF-I for 24 h. Fig 10B. Expression quantity of *keratin 26* was 0.00005124 × (p < 0.01), 0.00009017 × (p < 0.01), 0.0001944 × (p < 0.01) less in the groups which cells were treated with 10^−6^ g/L, 10^−5^ g/L, 10^−4^ g/L IGF-I than the group which cells were treated for 0 g/L IGF-I for 48 h. Fig 10C. Expression quantity of *keratin 26* was 0.00009369 × (p < 0.01), 0.00012564 × (p < 0.01), 0.00015526 × (p < 0.01) less in the groups which cells were treated with 10^−6^ g/L, 10^−5^ g/L, 10^−4^ g/L IGF-I than the group which cells were treated for 0 g/L IGF-I for 72 h.

After MT, FGF5 and IGF-I treated primary skin cells of goat, quantitative real-time PCR detection result showed that MT and FGF5 had positive regulation on *keratin 26* expression (Figs 8 and 9), IGF-I had negative regulation on *keratin 26* expression (Fig 10). When primary skin cells were treated with MT for 24 h, *keratin 26* relative expression quantity all increased in the groups treated with 0.02 g/L and 0.2 g/L MT, the effect of the 0.02 g/L MT was significant (p < 0.05), that of 0.2 g/L dose was extremely significant (p < 0.01), *keratin 26* relative expression quantity declined in the group treated with 1 g/L MT, the effect was significant (p < 0.05) (Fig 8A); when primary skin cells were treated with MT for 48 h, *keratin 26* relative expression quantity all increased, the effect of the 0.02 g/L MT was significant (p < 0.05), but that of 0.2 g/L and 1 g/L dose was not significant (p > 0.05) (Fig 8B); when primary skin cells were treated with MT for 72 h, *keratin 26* relative expression quantity all increased, the effect was all extremely significant (p < 0.01) (Fig 8C). *Keratin 26* relative expression quantity all increased in the groups treated with FGF5, the effect was significant in the groups treated with 10−^6^ g/L FGF5 for 24 h (p < 0.05), that of other dose was extremely significant (p < 0.01) (Fig 9). When primary skin cells were treated with IGF-I, *keratin 26* relative expression quantity all declined, the effect was all extremely significant (p < 0.01) (Fig 10).

**Fig 11:**
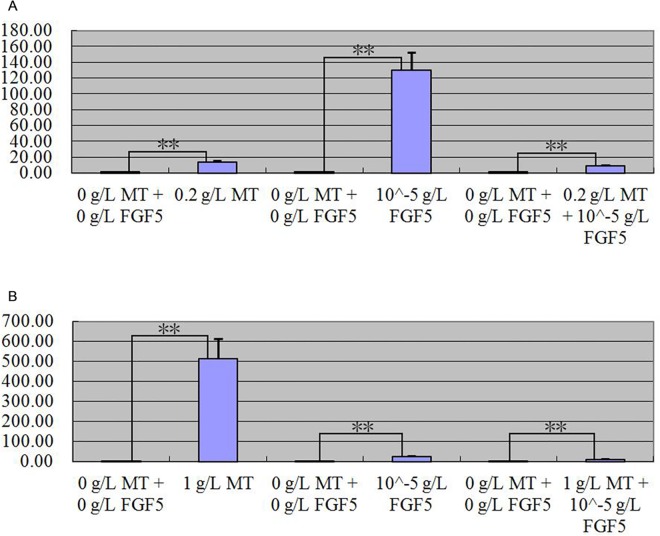
Expression quantity change of *keratin 26* after 0.2 g/L MT and 10^−5^ g/L FGF5 treatment together for 24 h or 1 g/L MT and 10^−5^ g/L FGF5 treatment together for 72 h was detected by quantitative real-time PCR. Fig 11A. Expression quantity of *keratin 26* was 13.21 × (p < 0.01) greater in the groups which cells were treated with 0.2 g/L MT than the group which cells were treated with 0 g/L MT for 24 h, 129.91 × (p < 0.01) greater in the groups which cells were treated with 10^−5^ g/L FGF5 than the group which cells were treated for 0 g/L FGF5 for 24 h, 9.09 × (p < 0.01) more in the group which cells were treated with 0.2 g/L MT and 10^−5^ g/L FGF5 together than the group which cells were treated with 0 g/L FGF5 and 0 g/L MT for 24 h. Fig 11B. Expression quantity of *keratin 26* was 513.36 × (p < 0.01) greater in the groups which cells were treated with 1 g/L MT than the group which cells were treated with 0 g/L MT for 72 h, 22.3 × (p < 0.01) greater in the groups which cells were treated with 10^−5^ g/L FGF5 than the group which cells were treated for 0 g/L FGF5 for 72 h, 9.74 × (p < 0.01) more in the group which cells were treated with 1 g/L MT and 10^−5^ g/L FGF5 together than the group which cells were treated with 0 g/L FGF5 and 0 g/L MT for 72 h.

**Fig 12:**
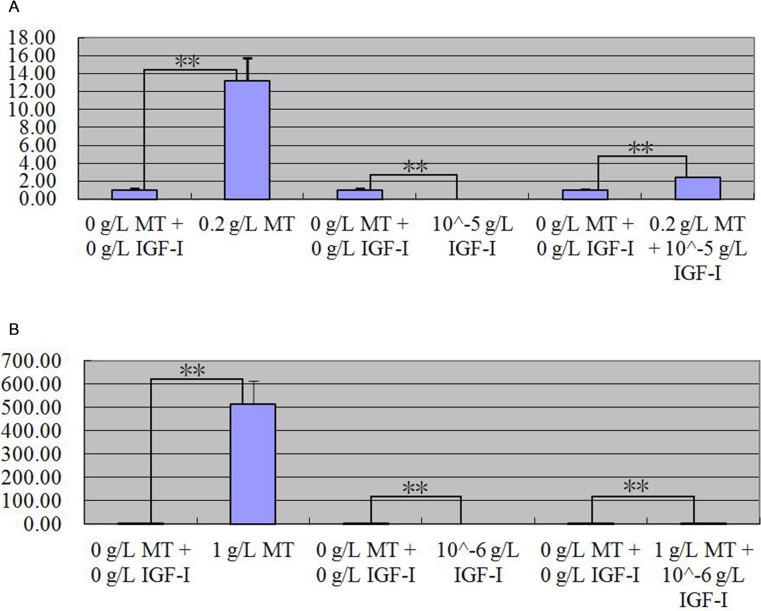
Expression quantity change of *keratin 26* after 0.2 g/L MT and 10^−5^ g/L IGF-I treatment together for 24 h or 1 g/L MT and 10^−6^ g/L IGF-I treatment together for 72h was detected by quantitative real-time PCR. Fig 12A. Expression quantity of *keratin 26* was 13.21 × (p < 0.01) greater in the groups which cells were treated with 0.2 g/L MT than the group which cells were treated with 0 g/L MT for 24 h, 0.0000083 × (p < 0.01) less in the groups which cells were treated with 10^−5^ g/L IGF-I than the group which cells were treated for 0 g/L IGF-I for 24 h, 2.41 × (p < 0.01) greater in the group which cells were treated with 0.2 g/L MT and 10^−5^ g/L IGF-I together than the group which cells were treated with 0 g/L IGF-I and 0 g/L MT for 24 h. Fig 12B. Expression quantity of *keratin 26* was 513.36 × (p < 0.01) greater in the groups which cells were treated with 1 g/L MT than the group which cells were treated with 0 g/L MT for 72 h, 0.00009369 × (p < 0.01) less in the groups which cells were treated with 10^−6^ g/L IGF-I than the group which cells were treated for 0 g/L IGF-I for 72 h, 3.92 × (p < 0.01) greater in the group which cells were treated with 1 g/L MT and 10^−6^ g/L IGF-I together than the group which cells were treated with 0 g/L IGF-I and 0 g/L MT for 72 h.

After MT and FGF5 treated primary skin cells of goat together, quantitative real-time PCR detection result indicated that *keratin 26* relative expression quantity significantly all increased in two groups that primary skin cells were treated with 0.2 g/L MT and 10−^5^ g/L FGF5 together for 24 h and 1 g/L MT and 10−^5^ g/L FGF5 together for 72 h (p < 0.01); compared with the group treated with MT or FGF5 respectively, the positive regulatory effect of MT and FGF5 on *keratin 26* all weakened (Fig 11). After MT and IGF-I treated primary skin cells of goat together, quantitative real-time PCR detection result indicated that *keratin 26* relative expression quantity all increased in two groups that primary skin cells were treated with 0.2 g/L MT and 10−^5^ g/L FGF5 together for 24 h and 1 g/L MT and 10−^6^ g/L FGF5 together for 72 h (p < 0.01); compared with the group treated with MT, the positive regulatory effect of MT on *keratin 26* weakened; compared with the group treated with IGF-I, the negative regulatory effect of IGF-I on *keratin 26* translated into positive regulation (Fig 12).

**Fig 13:**
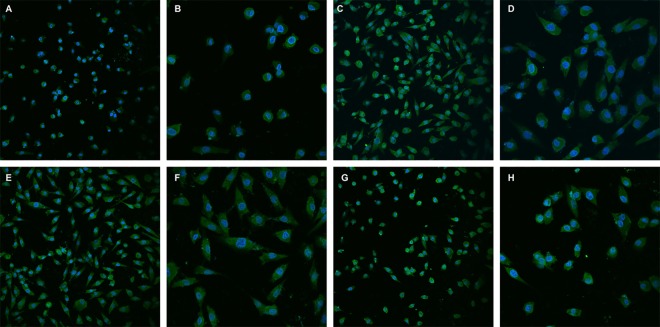
Skin cells which were treated with 0.2 g/L MT, 10^−5^ g/L FGF5, or 10^−6^ g/L IGF-I respectively for 72 h were observed under bright field of the laser scanning confocal microscope. Fig 13A. Skin cells which were treated without MT, FGF5 or IGF-I (25×). Fig 13B. Skin cells which were treated without MT, FGF5 or IGF-I (100×). Fig 13C. Skin cells which were treated with 0.2 g/L MT (25×). Fig 13D. Skin cells which were treated with 0.2 g/L MT (100×). Fig 13E. Skin cells which were treated with 10^−5^ g/L FGF5 (25×). Fig 13F. Skin cells which were treated with 10^−5^ g/L FGF5 (100×). Fig 13G. Skin cells which were treated with 10^−6^ g/L IGF-I (25×). Fig 13H. Skin cells which were treated with 10^−6^ g/L IGF-I (100×).

After MT, FGF5 and IGF-I treated primary skin cells of goat, gray analysis of western blot experiment results showed that keratin 26 expression quantity change was the greatest in the group treated with 0.02 g/L MT for 48 h, the change took second place and third place in the groups treated with 1 g/L MT for 48 h and 0.2 g/L MT for 72 h, but the cell status in the groups treated with 0.02 g/L and 1 g/L MT for 48 h was all not good, thus, treating primary skin cells of goat with 0.2 g/L MT for 72 h was the best condition for MT groups; in addition, treating primary skin cells of goat with 10−5 g/L FGF5 or 10−6 g/L IGF-I for 72 h were the best conditions for FGF5 or IGF-I groups (Table 4). After 0.2 g/L MT, 10−5 g/L FGF5 or 10−6 g/L IGF-I for 72 h treated primary skin cells of goat, immunofluorescence results indicated that keratin 26 expression all increased comparing with the negative control group (Fig 13).

**Table 4:** The results of gray analysis after treatment with MT, FGF5, IGF-I in immunofluorescence detection preliminary experiment.

Treatment concentrations	Treatment time		
	24 h	48 h	72 h
**NC**	1	1	1
**0.02 g/L MT treatment**	0.98	1.17	0.96
**0.2 g/L MT treatment**	0.96	0.96	1.03
**1 g/L MT treatment**	0.95	1.08	0.77
**10**^**−6**^ **g/L FGF5 treatment**	1.16	1.04	1.1
**10**^**−5**^ **g/L FGF5 treatment**	1.21	1.15	7.79
**10**^**−4**^ **g/L FGF5 treatment**	1.01	2.41	8.26
**10**^**−6**^ **g/L IGF-I treatment**	1.12	1.89	7.32
**10**^**−5**^ **g/L IGF-I treatment**	1.06	2.27	0.97
**10**^**−4**^ **g/L IGF-I treatment**	1.21	1.08	1.06

## Discussion

### Location and expression of *keratin 26* and keratin 26

*Keratin 26* expression of Liaoning cashmere goat had been located through quantitative real-time PCR before, the results showed that *keratin 26* was specifically expressed in skin [12]; then our study further found that *keratin 26* was specifically expressed in cortex layer of skin hair follicles through in situ hybridization (Figs 1 and 2), previous research indicated that *keratin 26* of human was specifically expressed in inner root sheath of skin hair follicles [2], the difference of *keratin 26* expression location between cashmere goat and human might be resulted from rather distant phylogenetic relationships [12]. Immunohistochemistry experiment results showed that keratin 26 was specifically expressed in inner root sheath and outer root sheath (Figs 3 and 4), we could find that expression locations between *keratin 26* and keratin26 was not consistent, it illustrates that *keratin 26* is transcribed and translated into keratin 26 first, then keratin 26 is transferred to inner root sheath and outer root sheath and exerts its function.

We know that the growth cycles of hair follicles and cashmere are similar, cashmere growth is strong during hair follicle anagen is strong, however, cashmere growth rate slowes during hair follicle catagen, cashmere almost does not grow during hair follicle telogen. Quantitative real-time PCR results showed that there basically was no difference between primary and secondary follicles of *keratin 26* expression quantity during anagen (Fig 5A), *keratin 26* expression proportion in secondary follicles increased significantly comparing with primary follicles from anagen to catagen of hair follicles (Fig 5B), cashmere growth rate slowed with growth cycle of hair follicles entering into catagen, however, *keratin 26* expression quantity in secondary follicles produced cashmere increased significantly, thus we speculate that *keratin 26* has negative regulation effect on cashmere growth, and is related to entering into catagen of hair follicles; because primary and secondary follicles couldn’t be distinguished clearly during telogen of hair follicles, we detected *keratin 26* expression of skin, comparing with the two former periods, *keratin 26* expression quantity increased substantially during telogen (Fig 5C), cashmere growth almost stopped during this period, we can find that *keratin 26* may be related to entering into telogen of hair follicles, and it further illustrates that *keratin 26* can inhibit cashmere growth.

### *Noggin* expression interference and *keratin 26* overexpression

*Noggin* expression interference experiment showed that *keratin 26* relative expression quantity declined when *Noggin* relative expression quantity declined (Fig 7A), on the contrary, *keratin 26* overexpression experiment indicated that *Noggin* relative expression quantity increased when *keratin 26* relative expression quantity increased (Fig 7B), the relative expression quantity change tendency of *Noggin* was consistent with that of *keratin 26*, *Noggin* and *keratin 26* may be correlative genes. We know that BMP can inhibit cell development of hair follicles [46], Noggin, as BMP signaling pathway antagonist, could prevent the combination between BMP2, BMP4 and their receptors through combining BMP2 and BMP4 in BMP signaling pathway, and then the signaling pathway was blocked [47], thus we speculate that *keratin 26* may locate in the upstream of *Noggin* and BMP signaling pathway. When *Noggin* expression is weaker, BMP signaling pathway is activated and exerts the function inhibiting cell development of hair follicles and cashmere formation normally, upstream *keratin 26* expression quantity declined after receiving the feedback of *Noggin* expression quantity; *Noggin* expression quantity increased after *keratin 26* overexpression, Noggin inhibits BMP signaling pathway transmission, and then *keratin 26* may inhibit growth and development of cashmere through other pathways or block it directly, by this taken, *keratin 26* and BMP signaling pathway are mutual antagonistic pathways inhibiting growth and development of cashmere, it also means that *keratin 26* expression is blocked when BMP signaling pathway is activated, on the contrary, BMP signaling pathway is blocked when *keratin 26* expression quantity increases.

### MT, FGF5, and IGF-I effects on expression of *keratin 26* and keratin 26

After MT, FGF5 and IGF-I treated primary skin cells of goat, *keratin 26* relative expression quantity increased in general in the groups treated with MT and FGF5 (Figs 8 and 9), declined in general in the group treated with IGF-I (Fig 10). Next, we wanted to know more about the effect of treating primary skin cells of goat with MT and FGF5 or MT and IGF-I together on *keratin 26* expression quantity, thus we screened the best conditions for MT, FGF5 and IGF-I groups first. In MT group, *keratin 26* relative expression quantity change was the greatest after treating with 1 g/L MT for 72 h, and the difference was extremely significant (p < 0.01), therefore it was the best condition for MT group (Fig 8); in FGF5 and IGF-I groups, *keratin 26* relative expression quantity change was the greatest after treating with 10−^5^ g/L FGF5 or IGF-I for 24 h, and the difference was all extremely significant (p < 0.01), thus treating with 10−^5^ g/L FGF5 or IGF-I for 24 h was the best condition for FGF5 or IGF-I group (Figs 9 and 10). In the experiment treating primary skin cells of goat with MT and FGF5 together, because the best conditions for MT and FGF5 groups were not consistent, first based on the best treating time for MT group (72 h), 10−^5^ g/L FGF5 was the best treating condition (p < 0.01); next based on the best treating time for FGF5 group (24 h), 0.2 g/L MT was the best treating condition (p < 0.01), thus primary skin cells of goat were treated with 1 g/L MT and 10−^5^ g/L FGF5 together for 72 h or 0.2 g/L MT and 10−^5^ g/L FGF5 together for 24h. In the experiment treating primary skin cells of goat with MT and IGF-I together, because the best conditions for MT and IGF-I groups were not consistent, first based on the best treating time for MT group (72 h), 10−^6^ g/L IGF-I was the best treating condition (p < 0.01); next based on the best treating time for IGF-I group (24 h), 0.2 g/L MT was the best treating condition (p < 0.01), thus primary skin cells of goat were treated with 1 g/L MT and 10−^6^ g/L IGF-I together for 72 h or 0.2 g/L MT and 10−^5^ g/L IGF-I together for 24h. Whether primary skin cells of goat were treated with 0.2 g/L MT and 10−^5^ g/L FGF5 together for 24 h or 1 g/L MT and 10−^5^ g/L FGF5 together for 72 h, *keratin 26* relative expression quantity extremely significantly all increased (p < 0.01) (Fig 11), compared with the group treated with MT or FGF5 respectively, the positive regulatory effect of MT and FGF5 on *keratin 26* declined (Fig 11), it illustrates that FGF5 can weaken the promoting effect of MT on *keratin 26* expression quantity, MT can also weaken the promoting effect of FGF5 on *keratin 26* expression quantity; so MT and FGF5 can weaken their regulatory effect on *keratin 26* each other. After primary skin cells of goat were treated with 0.2 g/L MT and 10−^5^ g/L IGF-I together for 24 h or 1 g/L MT and 10−^6^ g/L IGF-I together for 72 h, *keratin 26* relative expression quantity significantly all increased (p < 0.05) (Fig 12), compared with the group treated with MT, the positive regulatory effect of MT on *keratin 26* declined (Fig 12), it illustrates that IGF-I can weaken the promoting effect of MT on *keratin 26* expression quantity; compared with the group treated with IGF-I, the negative regulatory effect of IGF-I on *keratin 26* translated into positive regulation (Fig 12), it illustrates that MT plays a leading role when primary skin cells of goat were treated with MT and IGF-I together.

After MT, FGF5 and IGF-I treated primary skin cells of goat, we also observed and detected keratin 26 expression change through immunofluorescence technology, the results showed that keratin 26 expression quantity all increased, and expression location keratin all transferred from perinuclear space in the negative group to endochylema and had the trend secreted out of the cells in the groups treated with MT, FGF5 and IGF-I (Fig 13). In IGF-I group, *keratin 26* and keratin 26 had the opposite change tendency, previous study found that IGF-I was the key factor affecting animal hair development and periodic change of hair follicle [26, 48], and could promote the growth and development of secondary follicles in cashmere goat [49], *keratin 26* expression quantity declined, but keratin 26 expression quantity increased, it might be resulted from the inhabitation of keratin 26 degradation after treating with IGF-I, so keratin 26 expression quantity behaved as increase, keratin 26 degradation was blocked, it couldn’t play the role on negative regulation effect on cashmere growth normally, and then promoted cashmere growth and development indirectly, therefore we speculate that keratin 26 can exert the function inhibiting cashmere growth through its degradation, and IGF-I can promote cashmere growth and development through regulating *keratin 26* expression negatively and inhibiting keratin 26 degradation. In FGF5 group, *keratin 26* and keratin 26 had the same change tendency, the expression quantity all increased, some scholars found that FGF5 was involved in the regulation that hair follicles transformed from anagen into telogen before, FGF5 protein could induce catagen activation and inhibit hair growth [50], *keratin 26* also has negative regulation effect on cashmere growth, thus we think that FGF5 can play the role on regulating hair growth through promoting *keratin 26* expression and keratin 26 degradation (Keratin 26 expression quantity increased in performance, but *keratin 26* expression quantity also increased, thus keratin 26 expression quantity could also show an relative increase in the case of protein degradation quantity increase). (3) In MT group, *keratin 26* expression quantity all increased significantly after treating primary skin cells of goat with 0.02 g/L MT for 24 h, 48 h or 72 h, and the difference gradually enhanced with the increase on treating time, *keratin 26* expression quantity change was stable under this treating concentration; *keratin 26* expression quantity all increased significantly after treating primary skin cells of goat with 0.2 g/L MT for 24 h or 72 h, and the difference was all extremely significant, however, *keratin 26* expression quantity also increased after treating primary skin cells of goat with 0.2 g/L MT for 48 h, the difference was not significant, the stability of *keratin 26* expression quantity change was general under this treating concentration, but *keratin 26* expression quantity all increased generally; *keratin 26* expression quantity decreased firstly, then increased after treating primary skin cells of goat with 1 g/L MT for 24 h, 48 h or 72 h, *keratin 26* expression quantity change was very unstable under this treating concentration; on the whole, MT could up-regulated *keratin 26* expression, and promote keratin 26 expression, *keratin 26* and keratin 26 had the same change tendency, previous researches found that MT could affect cashmere growth of goat and induce cashmere production in advance [51–52], some scholars also found that MT could promote the transformation from telogen to anagen [17], our previous experiment showed that *keratin 26* was related to entering into catagen and telogen of hair follicles and promote the change of hair follicle cycle, thus we speculate that keratin 26 itself can promote the end of catagen and telogen producing less cashmere rapidly, and then shorten hair follicle cycle and induce cashmere production in advance, we can find that MT can induce cashmere production in advance through promoting *keratin 26* and keratin 26 expression.

## Conclusions

In conclusion, keratin 26 has negative regulation effect on cashmere growth and is related to entering into catagen and telogen of hair follicles. *Keratin 26* and BMP signaling pathway are mutual antagonistic pathways inhibiting growth and development of cashmere, it also means that *keratin 26* expression is blocked when BMP signaling pathway is activated, on the contrary, BMP signaling pathway is blocked when *keratin 26* expression quantity increases. In addition, MT and FGF5 have the positive regulatory effect on *keratin 26* and keratin 26, MT and FGF5 can weaken their regulatory effect on *keratin 26* each other; IGF-I has the negative regulatory effect on *keratin 26* and the positive regulatory effect on keratin 26, IGF-I can weaken the regulatory effect of MT on *keratin26*, however, MT still plays a leading role when primary skin cells of goat were treated with MT and IGF-I together. Keratin 26 is one of important pathways that MT induces cashmere production in advance and FGF5 regulates cashmere growth and IGF-I promoted cashmere growth and development. How to use of the antagonism between *keratin 26* and BMP signaling pathway and the mechanism that MT, FGF5 and IGF-I affect *keratin 26* and keratin 26 more effectively in complicated body of Liaoning cashmere goat, and then promote the quality and yield of cashmere, it is the problem that needs to be further researched and solved.

## Supporting Information

S1 Fig Skin cells before *Noggin* expression interference and *keratin 26* overexpression were observed under the microscope. (TIF)

S2 Fig Skin cells in blank control group, negative control group and experimental group were observed under the microscope in *Noggin* expression interference experiment. (TIF)

S3 Fig Skin cells in blank control group, negative control group and experimental group were observed under the microscope in *keratin 26* overexpression experiment. (TIF)

S4 Fig Skin cells before treating with MT, FGF5 or IGF-I respectively were observed under the microscope in the immunofluorescence experiment. (TIF)
