# Supplementary figures and images for: The Study on Biological Function of Keratin 26, a Novel Member of Liaoning Cashmere Goat Keratin Gene Family
Source: PLoS One. 2016 Dec 20;11(12):e0168015. doi: 10.1371/journal.pone.0168015 (PMC5173340; doi:10.1371/journal.pone.0168015)

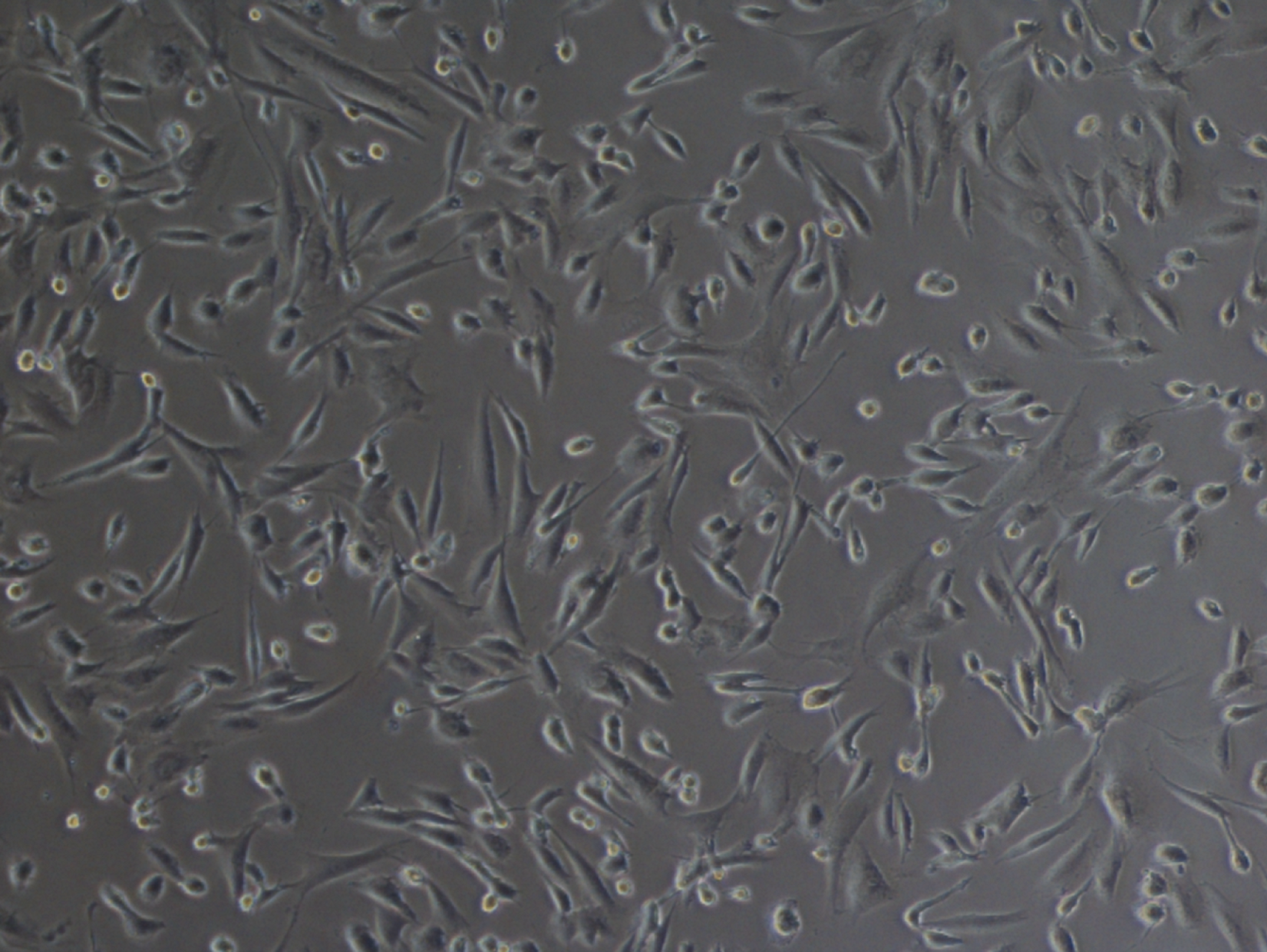

Supplement: S1 Fig — (TIF) [file pone.0168015.s001.tif]

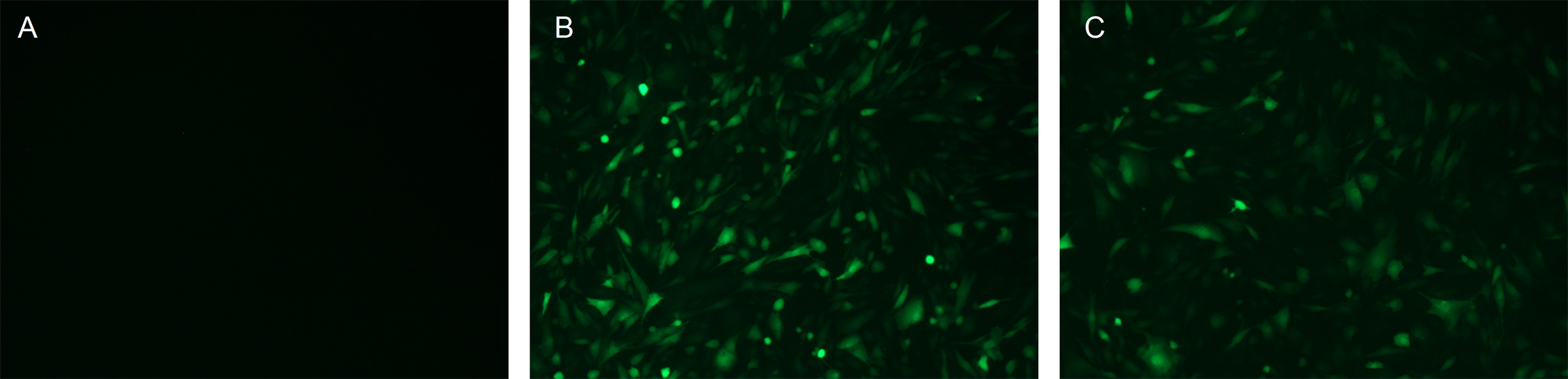

Supplement: S2 Fig — (TIF) [file pone.0168015.s002.tif]

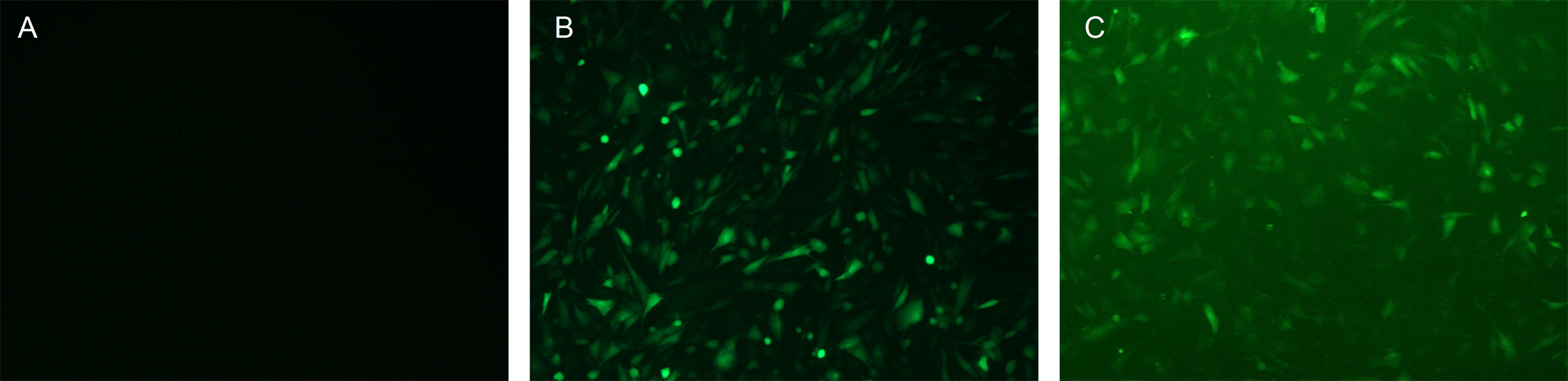

Supplement: S3 Fig — (TIF) [file pone.0168015.s003.tif]

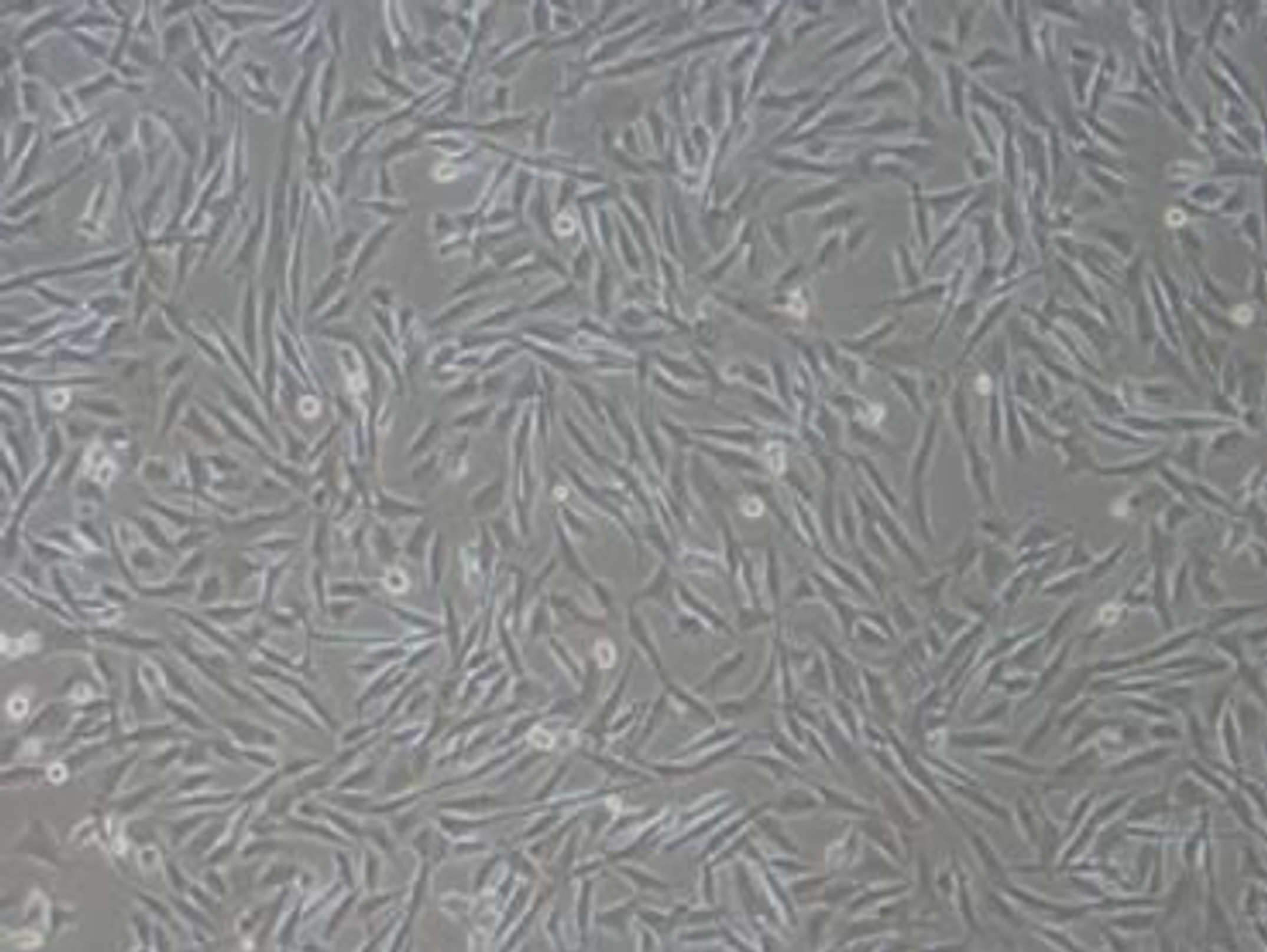

Supplement: S4 Fig — (TIF) [file pone.0168015.s004.tif]
